# STEMI under fire: evaluating management and challenges in a warzone amidst the 2023 Israeli conflict

**DOI:** 10.1186/s12913-025-12809-3

**Published:** 2025-05-08

**Authors:** Vladimir Zeldetz, Sagi Shashar, Carlos Cafri, David Shamia, Tzachi Slutsky, Noa Fried Regev, Naif Abu Abed, Dan Schwarzfuchs

**Affiliations:** 1 Department of Emergency Medicine, Soroka University Medical Center, Beer-Sheva, Israel; 2 Clinical Research Center, Soroka University Medical Center and Faculty of Health Sciences, Ben-Gurion University of the Negev, P.O. Box 151, Beer-Sheva, 84101 Israel; 3 Department of Cardiology, Soroka University Medical Centre, Beer Sheva, Israel; 4 Hospital Administration, Soroka University Medical Center, Beer Sheva, Israel

**Keywords:** Myocardial infarction, Late arrival, War, Conflict-related stress, Healthcare delivery in war zones

## Abstract

**Background:**

Previous studies highlight the impact of conflict and war on cardiovascular health, suggesting increased incidence of events like STEMI due to heightened stress and healthcare disruptions. However, specific data on STEMI management and outcomes during active conflicts remain limited. This study assesses the impact of the October 2023 war in Israel on STEMI incidence, late arrivals, and the potential correlation with the intensity of rocket fire.

**Methods:**

This retrospective cohort study was conducted at Soroka University Medical Center, Beer Sheva, Israel, from 2021 to 2023. Data from patients admitted with STEMI during a two-month period (October 7 to December 7) across these years were analyzed. Patient demographics, arrival characteristics, clinical and PCI characteristics, and outcomes were compared across pre-war (2021–2022) and war (2023) periods. Multivariable logistic regression identified predictors of late arrivals, and Spearman correlation assessed the relationship between rocket attacks and STEMI cases and late arrivals.

**Results:**

The study included 193 STEMI patients (83.4% male, average age 62.87 years). A significant increase in late arrivals was observed during the war period (28.8% in 2023 vs. 10.2% pre-war, *p* = 0.002). Ambulance arrivals decreased (34.8% in 2023 vs. 59.1% pre-war), while referrals from emergency centers increased (57.6% in 2023 vs. 25.2% pre-war, *p* < 0.001). Clinical characteristics and PCI outcomes including time metrics such as door-to-balloon (D2B) and pain-to-balloon (P2B), showed no significant differences between the periods. The period of war was a significant predictor of late arrivals (AdjOR 3.12, 95% CI 1.29–7.85, *p* = 0.013). Correlation analysis between rocket attacks and STEMI cases was not statistically significant.

**Conclusions:**

While hospital care remained robust, there was a marked increase in late arrivals and patients coming from emergency centers, indicating delays in seeking medical attention and fear of going directly to the hospital. These findings highlight the need for targeted patient education to ensure prompt care during conflicts and improve confidence in hospital safety and availability.

## Introduction

ST-elevation myocardial infarction (STEMI) is a severe form of acute coronary syndrome requiring urgent medical intervention to restore blood flow and minimize cardiac damage [1]. Numerous studies have highlighted the impact of various external stressors on cardiovascular health, particularly during periods of conflict and war [2, 3]. Previous research indicates that the incidence of cardiovascular events, including STEMI, may increase during such times due to heightened stress, delayed medical response, and disruptions in healthcare services [3]. Additionally, these events can influence the time to primary percutaneous coronary intervention (PCI), clinical outcomes, and the number of late arrivals at medical facilities [4].

The war that began on October 7, 2023, in Israel, has been characterized by intense rocket fire and military actions, profoundly affecting daily life and healthcare delivery in affected areas [5]. Soroka University Medical Center (SUMC), located in Beer Sheva, Israel, is the primary tertiary healthcare provider for the Negev region and the largest hospital in the war zone [6]. The Negev region has a population of about 1 million people, approximately 30% of whom are Bedouin Arabs, spread across an area of roughly 13,000 square kilometers [7]. This institution has been at the forefront of managing the influx of both civilian and military casualties, providing a unique context to study the impact of war on STEMI management [8].

While existing literature provides insights into the broader implications of war on health, specific data on STEMI incidence, management practices, and outcomes during active conflict remain limited. This study aims to fill this gap by assessing the impact of the war on the incidence of STEMI, the prevalence of late arrivals, and the potential correlation between the number of rockets launched and these health outcomes, during a two-month period from October 7 to December 7 across the years 2021–2023. We aim to provide valuable insights into the challenges and adaptations in STEMI management during wartime, contributing to the broader understanding of healthcare delivery under extreme conditions. This study’s findings could inform future strategies to improve cardiovascular care during conflicts and similar crises.

## Methods

### Study design and population

The study was a retrospective cohort study, conducted at the at SUMC, Beer Sheva, Israel, between the years 2021–2023. The study encompassed all cases of STEMI admitted to our institution over a two-month period from October 7 to December 7 for the years 2021–2023. These months were selected because they represented the most intense period of rocket fire in the region, significantly disrupting daily life and healthcare delivery, thus providing a true reflection of a warzone environment. Only patients presenting with new-onset STEMI from outside the hospital were included—either those who arrived at the emergency department (ED) or were diagnosed in the field by emergency medical services (EMS) and transferred directly to the intensive cardiac care unit (ICCU) for primary PCI. Patients who developed STEMI during hospitalization for other conditions, those under 18 years of age, transfers from other hospitals, helicopter evacuations, and individuals with foreign citizenship were excluded (due to limited availability of background medical information in the national systems and the inability to follow their post-discharge outcomes such as mortality).

### Data collection

Data for the study were extracted and reviewed from computerized records at SUMC and included demographic characteristics, arrival characteristics, clinical and PCI characteristics and outcomes. The institutional ‘Helsinki’ review board approved the study (0298-23-SOR), informed consent was not required as the study was retrospective, and the data were kept anonymized and encoded. There were no missing data in the study, as all information was manually extracted and verified from complete electronic medical records.

The demographic characteristics collected included gender, age, ethnicity, Charlson Comorbidity Index (CCI), chronic ischemic heart disease (CIHD), diabetes mellitus (DM), hypertension (HTN), dyslipidemia, and smoking status. Ethnicity classification (classified as Bedouin or Jewish) was based on a combination of indicators, including the patient’s first and last names, which typically align with either Arabic or Hebrew linguistic patterns. In cases where names were not definitive, supplementary information such as residential area and the affiliated primary care clinic was considered, as certain regions and clinics predominantly serve specific ethnic communities [9].

Arrival characteristics documented the time of arrival, categorized into within 12 h, between 12-48 h, and above 48 h from symptom onset. This classification corresponds to acute STEMI (within 12 h), evolved STEMI (12–48 h), and recent STEMI (over 48 h), in line with ESC 2017 guidelines [10]. Although primary PCI is recommended within 12 h, patients arriving later may still undergo urgent intervention based on clinical indications such as ongoing chest pain, hemodynamic instability, or malignant arrhythmias. The mode of arrival included whether the patient arrived by ambulance ordered by the patient, referral from an emergency medical center, or self-arrival. Emergency medical centers encompassed outpatient clinics, small local emergency centers, and private physicians, which had limited capabilities for monitoring, immediate response to complications of an acute myocardial infarction, and lacked facilities for definitive treatment. Additionally, it was noted whether patients arrived at the ED or directly to the ICCU. Clinical characteristics upon arrival included the assessment of left ventricular function (LVF), which was categorized into mild LV dysfunction, moderate LV function, normal/preserved LV function, and severe LV function. The presence of arrhythmias was documented as none, ventricular tachycardia/ventricular fibrillation (VT/VF), and complete atrioventricular block (Complete AV block). Instances of cardiogenic shock prior to PCI were also noted. PCI characteristics included spontaneous reperfusion, the use of tissue plasminogen activator (TPA), and various timing metrics collected for patients arriving within 12 h of symptom onset, including the time from first medical contact (FMC), pain to door (P2D), door to needle (D2N), door to balloon (D2B), and pain to balloon (P2B).

### Outcomes

The outcomes measured were in-hospital death, death within 30 days, death within 30–90 days, death within 90–180 days, and length of stay in the intensive cardiac care unit (LOS ICCU).

### Statistical analysis

Categorical variables were presented as frequencies and percentages, while continuous variables were assessed for normality using the Shapiro-Wilk test and visual inspection of histograms; variables with a normal distribution were presented as means with standard deviations (SD), while non-normally distributed variables were reported as medians with interquartile ranges (IQR). Demographic characteristics, arrival characteristics, clinical characteristics upon arrival, and PCI characteristics were compared across the years 2021, 2022, and 2023, as well as between the pre-war period (2021 + 2022) and the war period (2023). For comparisons across the three years (2021, 2022, and 2023), categorical variables were analyzed using the Chi-squared test, and continuous variables were analyzed using one-way ANOVA for parametric data and the Kruskal-Wallis test for non-parametric data. For the comparisons between two groups (pre-war vs. war), categorical variables were compared using the Chi-squared test, and continuous variables were compared using the unpaired Student’s t-test for parametric data and the Mann-Whitney U test for non-parametric data.

Multivariable logistic regression models were used to identify predictors of late arrivals (defined as more than 12 h after symptom onset). The regression models included variables selected based on clinical relevance and results from univariate analyses. The results of the logistic regression analyses were presented as adjusted odds ratios (AdjORs) with 95% confidence intervals (95% CIs) and *p*-values.

The correlation between the number of rockets launched and the number of patients arriving with STEMI and late arrivals was assessed using the Spearman correlation method. This included lagged correlations with lag periods ranging from 1 to 7 days, rolling correlations using windows of 3, 5, 7, and 14 days, and cumulative correlations.

All statistical analyses were performed using Python 3.8.5 and RStudio 2024.04.2. Results were considered statistically significant when the *p*-value was less than 0.05.

## Results

### Overall population

The study included a total of 193 patients with STEMI admitted to Soroka University Medical Center between 2021 and 2023, after excluding 4 patients: 2 who were evacuated by helicopter (1 from each period) and 2 foreign citizens (1 from each period). Among these patients, 161 (83.4%) were male, with an average age of 62.87 years ± 11.90. The majority of the patients, 147 (76.2%), were Jewish. The median CCI was 2.00 [IQR 0.00, 9.00], indicating a moderate level of comorbid conditions. CIHD was present in 43 (22.3%) patients, while 78 (40.4%) had DM, 92 (47.7%) had HTN, 146 (75.6%) had dyslipidemia, and 97 (50.3%) were smokers (Table 1).

**Table 1:** Demographic characteristics, arrival characteristics, clinical characteristics upon arrival, and PCI characteristics of STEMI patients during 2021–2023

		Overall	2021	2022	2023	*P*-value	Before war (2021 + 2022)	*P*-value
	n	193	55	72	66		127	
Demographic characteristics and background diseases	Male gender, n (%)	161 (83.4)	47 (85.5)	55 (76.4)	59 (89.4)	0.109	102 (80.3)	0.16
	Age (mean (SD))	62.87 (11.90)	62.13 (12.71)	64.93 (11.32)	61.23 (11.69)	0.163	63.72 (11.97)	0.169
	Jew ethnicity, n (%)	147 (76.2)	46 (83.6)	51 (70.8)	50 (75.8)	0.244	97 (76.4)	1
	CCI (median [range])	2.00 [0.00, 9.00]	2.00 [0.00, 9.00]	3.00 [0.00, 7.00]	2.00 [0.00, 7.00]	0.243	2.00 [0.00, 9.00]	0.23
	CIHD, n (%)	43 (22.3)	10 (18.2)	14 (19.4)	19 (28.8)	0.289	24 (18.9)	0.166
	DM, n (%)	78 (40.4)	20 (36.4)	30 (41.7)	28 (42.4)	0.766	50 (39.4)	0.798
	HTN, n (%)	92 (47.7)	25 (45.5)	30 (41.7)	37 (56.1)	0.222	55 (43.3)	0.126
	Dyslipidemia, n (%)	146 (75.6)	34 (61.8)	57 (79.2)	55 (83.3)	0.016	91 (71.7)	0.106
	Smoking, n (%)	97 (50.3)	25 (45.5)	35 (48.6)	37 (56.1)	0.478	60 (47.2)	0.312
Arrival characteristics	Arrival time, n (%)					0.019		0.003
	Above 48 h	12 (6.2)	2 (3.6)	4 (5.6)	6 (9.1)		6 (4.7)	
	Between 12–48 h	20 (10.4)	3 (5.5)	4 (5.6)	13 (19.7)		7 (5.5)	
	Within 12 h	161 (83.4)	50 (90.9)	64 (88.9)	47 (71.2)		114 (89.8)	
	Late arrivals > 12 h, n (%)	32 (16.6)	5 (9.1)	8 (11.1)	19 (28.8)	0.004	13 (10.2)	0.002
	Arrival by, n (%)					< 0.001		< 0.001
	Ambulance ordered by patient	98 (50.8)	38 (69.1)	37 (51.4)	23 (34.8)		75 (59.1)	
	Referral by an emergency medical center	70 (36.3)	7 (12.7)	25 (34.7)	38 (57.6)		32 (25.2)	
	Self	25 (13.0)	10 (18.2)	10 (13.9)	5 (7.6)		20 (15.7)	
	Arrival to ED (not directly to ICCU) n (%)	92 (47.7)	27 (49.1)	35 (48.6)	30 (45.5)	0.905	62 (48.8)	0.77
Clinical characteristics upon arrival	LVF, n (%)					0.217		0.143
	Mild LV dysfunction	67 (34.7)	17 (30.9)	25 (34.7)	25 (37.9)		42 (33.1)	
	Moderate LV function	62 (32.1)	20 (36.4)	20 (27.8)	22 (33.3)		40 (31.5)	
	Normal/preserved LV function	27 (14.0)	8 (14.5)	7 (9.7)	12 (18.2)		15 (11.8)	
	Severe LV function	37 (19.2)	10 (18.2)	20 (27.8)	7 (10.6)		30 (23.6)	
	Arrythmia, n (%)					0.02		0.11
	None	164 (85.0)	51 (92.7)	57 (79.2)	56 (84.8)		108 (85.0)	
	VT/VF	19 (9.8)	2 (3.6)	13 (18.1)	4 (6.1)		15 (11.8)	
	Complete AV block	10 (5.2)	2 (3.6)	2 (2.8)	6 (9.1)		4 (3.1)	
	Cardiogenic shock prior PCI, n (%)	17 (8.8)	2 (3.6)	10 (13.9)	5 (7.6)	0.118	12 (9.4)	0.867
PCI characteristics	Spontaneous reperfusion, n (%)	25 (13.0)	5 (9.1)	13 (18.1)	7 (10.6)	0.258	18 (14.2)	0.635
	TPA, n (%)	2 (1.0)	0 (0.0)	0 (0.0)	2 (3.0)	0.143	0 (0.0)	0.221
	FMC (median [range])	90.00 [7.00, 682.00]	87.00 [7.00, 682.00]	120.00 [10.00, 651.00]	90.00 [15.00, 620.00]	0.487	91.50 [7.00, 682.00]	0.591
	P2D (median [range])	137.00 [29.00, 710.00]	123.50 [29.00, 704.00]	155.00 [30.00, 693.00]	130.00 [40.00, 710.00]	0.352	145.00 [29.00, 704.00]	0.645
	D2N (median [range])	45.00 [7.00, 360.00]	44.00 [7.00, 270.00]	50.00 [7.00, 134.00]	35.00 [10.00, 360.00]	0.686	50.00 [7.00, 270.00]	0.385
	D2B (median [range])	63.00 [9.00, 365.00]	67.00 [9.00, 280.00]	68.00[14.00, 164.00]	52.00 [20.00, 365.00]	0.603	67.50 [9.00, 280.00]	0.457
	P2B (median [range])	200.00 [84.00, 803.00]	195.00 [89.00, 803.00]	224.00 [84.00, 760.00]	200.00 [88.00, 470.00]	0.506	200.50 [84.00, 803.00]	0.542
Outcomes	In hospital death, n (%)	16 (8.3)	4 (7.3)	10 (13.9)	2 (3.0)	0.066	14 (11.0)	0.102
	Death within 30 days, n (%)	21 (10.9)	6 (10.9)	12 (16.7)	3 (4.5)	0.074	18 (14.2)	0.073
	Death within 30–90 days, n (%)	3 (1.6)	0 (0.0)	2 (2.8)	1 (1.5)	0.455	2 (1.6)	1
	Death within 90–180 days, n (%)	1 (0.5)	0 (0.0)	1 (1.4)	0 (0.0)	0.43	1 (0.8)	1
	LOS ICCU (median [range])	4.00 [1.00, 15.00]	4.00 [2.00, 11.00]	4.00 [1.00, 15.00]	4.00 [1.00, 9.00]	0.729	4.00 [1.00, 15.00]	0.834

This table presents the overall and year-by-year distribution of key variables including gender, age, ethnicity, comorbidities, arrival times, and clinical characteristics. *P*-values are provided to indicate the statistical significance of differences observed across the years

### Comparisons between years

When comparing demographic characteristics across the years 2021, 2022, and 2023, no significant differences were observed in terms of gender (85.5% in 2021, 76.4% in 2022, 89.4% in 2023, *p* = 0.109), age (62.13 ± 12.71 in 2021, 64.93 ± 11.32 in 2022, 61.23 ± 11.69 in 2023, *p* = 0.163), or ethnicity (83.6% in 2021, 70.8% in 2022, 75.8% in 2023, *p* = 0.244). However, a significant increase in dyslipidemia was noted in 2023 (83.3%) compared to 2021 (61.8%) and 2022 (79.2%) (*p* = 0.016).

**Fig. 1:**
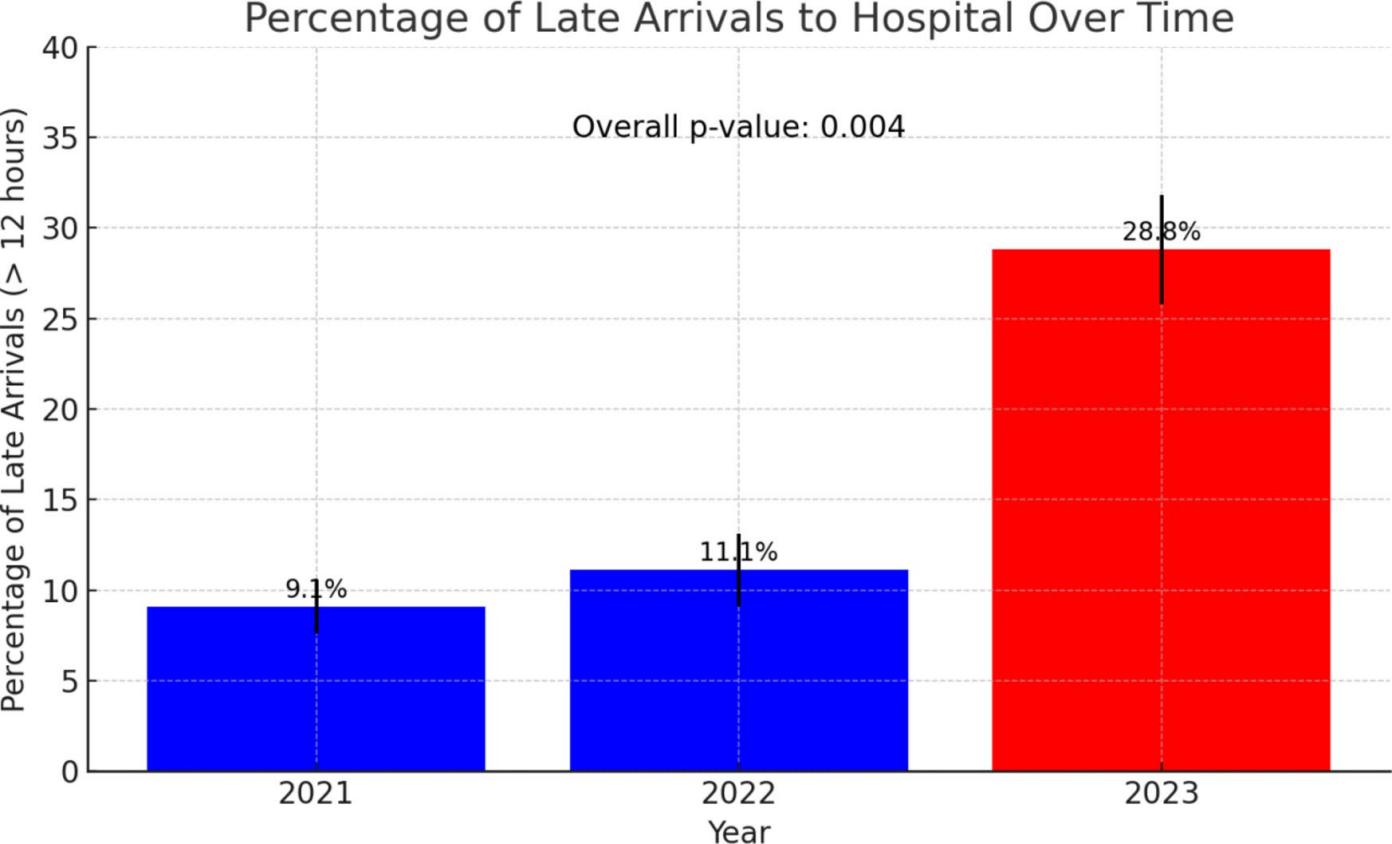
The graph illustrates the percentage of patients arriving late (more than 12 hours after symptom onset) to the hospital for the years 2021, 2022, and 2023. The error bars represent the standard deviations. The *p*-value for the comparison of late arrivals between the years is indicated as 0.004, demonstrating a statistically significant increase in late arrivals during 2023

Arrival times also showed significant variation, with a higher proportion of late arrivals (more than 12 h) in 2023 (19 patients, 28.8%) compared to 2021 (5 patients, 9.1%) and 2022 (8 patients, 11.1%) (*p* = 0.004), Fig. 1. The mode of arrival differed significantly across the years, with a decrease in ambulance arrivals (69.1% in 2021, 51.4% in 2022, 34.8% in 2023) and an increase in referrals from emergency medical centers (12.7% in 2021, 34.7% in 2022, 57.6% in 2023) and self-arrivals (18.2% in 2021, 13.9% in 2022, 7.6% in 2023) (*p* < 0.001).

**Fig. 2:**
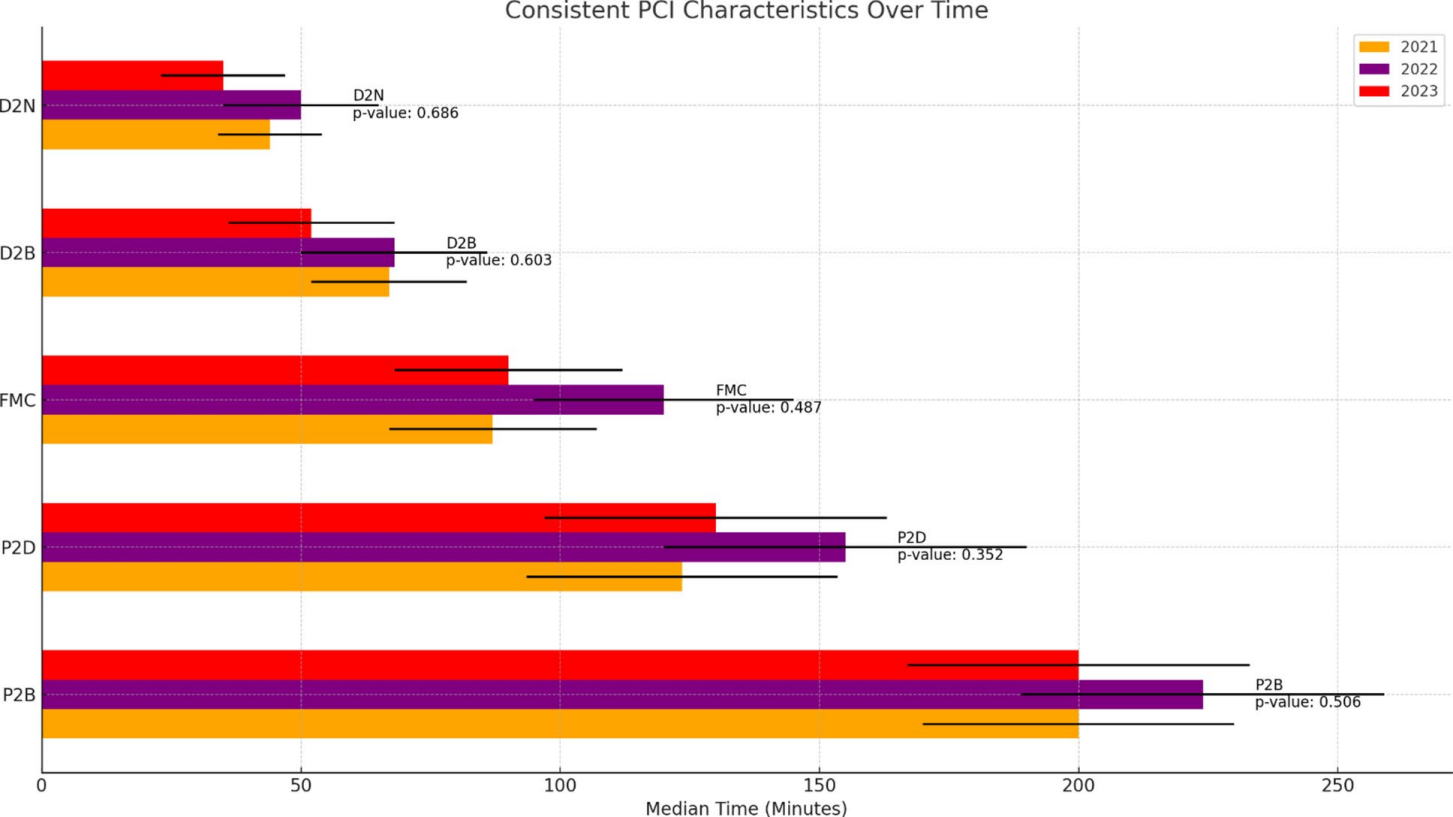
The graph shows the median times for key PCI characteristics across the years 2021, 2022, and 2023. The characteristics include: FMC (First Medical Contact), P2D (Pain to Door), D2N (Door to Needle), D2B (Door to Balloon), P2B (Pain to Balloon). The error bars represent the standard deviations for each measurement. *P*-values for the comparisons are displayed within the graph, indicating no statistically significant changes in these PCI characteristics over the observed years

No significant differences were observed in clinical characteristics upon arrival, including left ventricular function (LVF) and arrhythmias. The incidence of cardiogenic shock prior to PCI did not vary significantly between the years. Regarding PCI characteristics, no significant differences were observed in spontaneous reperfusion, use of tissue plasminogen activator (TPA), or various timing metrics such as FMC, P2D, D2N, D2B, and P2B, Fig. 2. The overall in-hospital mortality rate (8.3% overall, 7.3% in 2021, 13.9% in 2022, 3.0% in 2023, *p* = 0.066) and 30-day mortality rate (10.9% overall, 10.9% in 2021, 16.7% in 2022, 4.5% in 2023, *p* = 0.074) did not differ significantly across the years, although there was a trend towards lower mortality in 2023 (Table 1).

### Comparisons between pre-war and war periods

Comparing the pre-war period (2021 + 2022) with the war period (2023), significant differences were observed in arrival times and mode of arrival. Late arrivals were more frequent during the war period (19 patients, 28.8%) compared to the pre-war period (13 patients, 10.2%) (*p* = 0.002). Additionally, there was a notable shift in the mode of arrival, with fewer patients arriving by ambulance (59.1% pre-war vs. 34.8% war) and more being referred by emergency medical centers (25.2% pre-war vs. 57.6% war) or arriving by themselves (15.7% pre-war vs. 7.6% war) during the war (*p* < 0.001).

Clinical characteristics and PCI characteristics did not show significant differences between the two periods. There were no significant differences in in-hospital mortality (11.0% pre-war vs. 3.0% war, *p* = 0.102) or 30-day mortality (14.2% pre-war vs. 4.5% war, *p* = 0.073) (Table 1).

### Predictors of late arrivals

Multivariable logistic regression analysis identified the period of war as a significant predictor of late arrivals, with patients being more than three times as likely to arrive late during the war compared to routine periods (AdjOR 3.12, 95% CI 1.29–7.85, *p* = 0.013). Other significant predictors included arrival by referral from an emergency medical center (AdjOR 7.26, 95% CI 2.53–24.50, *p* < 0.001) and self-arrival (AdjOR 9.48, 95% CI 2.50–38.91, *p* = 0.001). Gender, age, ethnicity, and CCI were not significant predictors of late arrivals (Table 2).

**Table 2:** Predictors of late arrivals among STEMI patients; multivariable logistic regression

	Late arrivals		
*Predictors*	*Odds Ratios*	*CI*	*p*
(Intercept)	0.02	0.00–0.69	0.033
War vs. routine	3.12	1.29–7.85	0.013
Gender [Male]	0.78	0.25–2.65	0.674
Age	0.98	0.93–1.04	0.563
Ethnicity [Jew]	2.46	0.89–7.82	0.100
CCI	1.38	0.95–1.98	0.078
Arrival by [Referral by an emergency medical center]	7.26	2.53–24.50	< 0.001
Arrival by [Self]	9.48	2.50–38.91	0.001

This table shows the odds ratios, confidence intervals, and *p*-values for predictors of late arrivals (more than 12 h after symptom onset) to the hospital. Variables include the effect of war vs. routine periods, gender, age, ethnicity, comorbidity index (CCI), and mode of arrival

### Correlation between rockets and STEMI/late arrivals

**Fig. 3:**
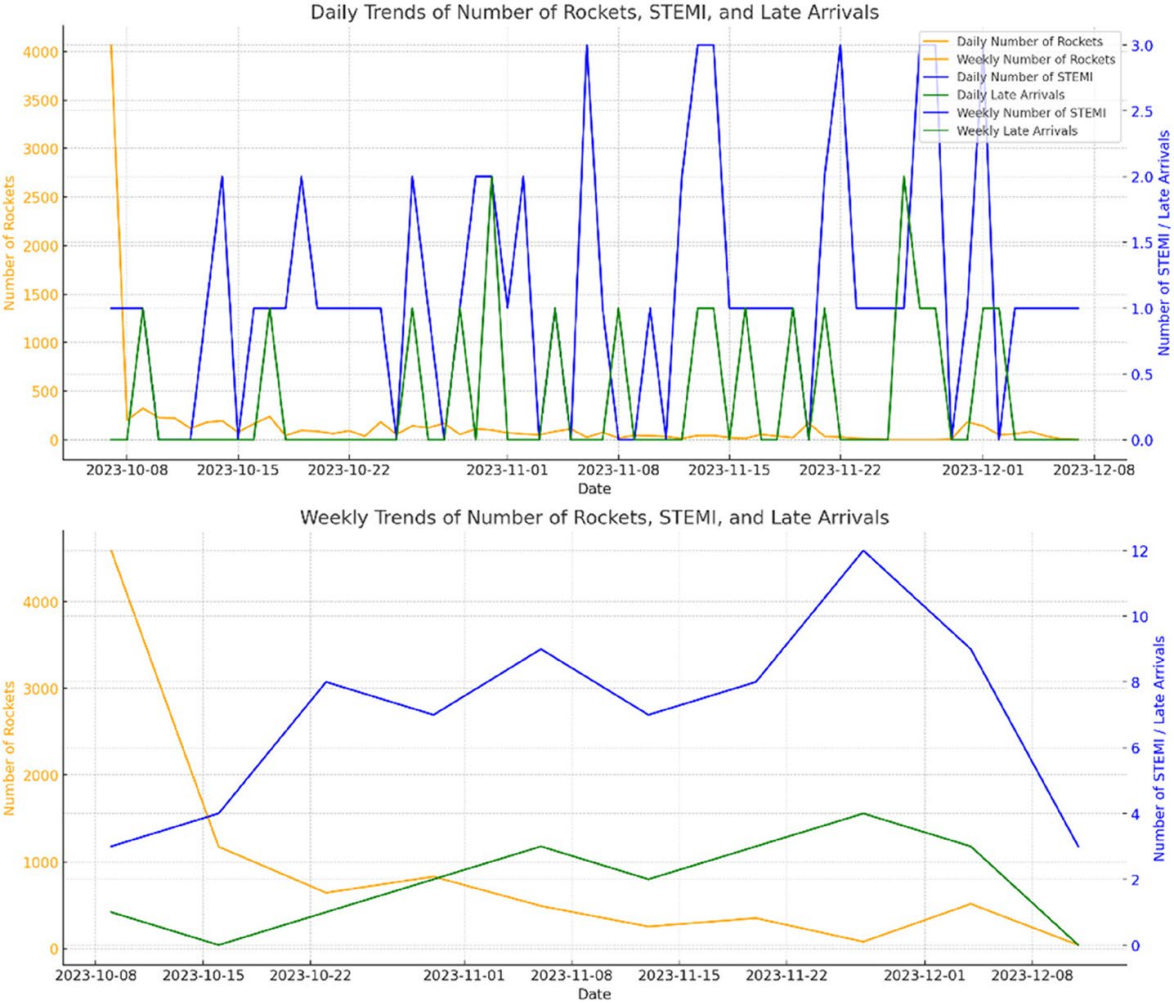
Shows the daily and weekly trends for the number of rockets, STEMI cases, and late arrivals. The dates are plotted in the x-axis and the number of rockets is plotted on the primary y-axis in orange. The number of STEMI cases and late arrivals are plotted on the secondary y-axis in blue and green, respectively

Overall, while the correlations suggested some potential relationships between rocket attacks and health outcomes, they were not statistically significant (Table 3; Fig. 3). The analysis revealed a positive correlation between the number of rockets 2 days before and the number of late arrivals (Spearman correlation = 0.109), although this was not statistically significant (*p* = 0.439). A slight positive correlation was observed with a 3-day rolling window (Spearman correlation = 0.083, *p* = 0.558). The cumulative sum of rockets showed a positive correlation with daily late arrivals (Spearman correlation = 0.108, *p* = 0.443) and with daily STEMI cases (Spearman correlation = 0.126, *p* = 0.372).

**Table 3:** Correaltion between number of rockets launtered and number of patients arrived with STEMI and late arrivals

Type	Window (days)	Late arrivals		STEMI arrivals	
		Correlation	*P*-value	Correlation	*P*-value
Lagged	1	-0.053	0.702	-0.060	0.666
Lagged	2	0.109	0.439	-0.046	0.735
Lagged	3	-0.054	0.697	-0.165	0.218
Lagged	4	-0.054	0.697	-0.158	0.239
Lagged	5	-0.041	0.759	-0.185	0.167
Lagged	6	-0.083	0.559	-0.064	0.646
Lagged	7	-0.069	0.619	0.050	0.711
Rolling	3	0.083	0.558	-0.039	0.772
Rolling	5	-0.091	0.519	-0.178	0.185
Rolling	7	-0.104	0.458	-0.081	0.571
Rolling	14	-0.110	0.434	-0.095	0.502
Cumulative		0.108	0.443	0.126	0.372

Table 3 presents the correlation analysis between the number of rockets and two health outcomes: late arrivals and STEMI, computed using the Spearman method. The table includes three types of correlations: Lagged Correlations, with lag periods ranging from 1 to 7 days, Rolling Correlations using rolling windows of 3, 5, 7, and 14 days, and Cumulative Correlations using the cumulative number of rockets and daily late arrivals/STEMI events. Each row in the table provides the correlation coefficient and the corresponding *p*-value

## Discussion

The present study provides a comprehensive analysis of the impact of the ongoing conflict, specifically the war that began on October 7, 2023, on the management and outcomes of STEMI at SUMC in Beer Sheva, Israel. The study found a significant increase in late arrivals (more than 12 h after symptom onset) during the war period, a marked decrease in ambulance arrivals, and no significant differences in clinical characteristics upon arrival or PCI outcomes between the pre-war and war periods. Furthermore, there was no significant correlation between the number of rockets launched and the number of STEMI cases or late arrivals.

These results highlight the considerable impact of conflict on healthcare delivery, particularly in acute cardiovascular care. The increase in late arrivals underscores the challenges patients face in accessing timely medical care during wartime. Previous research supports this, indicating that external stressors and disruptions in healthcare services during conflicts can delay medical response times and negatively impact patient outcomes [11, 12]. The significant rise in late arrivals during the war period points to the urgent need for feasible and context-specific strategies to ensure timely access to care even during conflicts.

### Ambulance use and referral patterns during conflict

The decrease in ambulance arrivals during the war, accompanied by a substantial increase in referrals from emergency medical centers and self-arrivals, suggests that many patients were hesitant to use ambulance services due to safety concerns or limited availability during the conflict. These emergency medical centers—comprising small local urgent care facilities —do not possess the necessary infrastructure for comprehensive cardiac care, such as continuous monitoring, immediate response to complications, or catheterization capabilities. Despite this, a notable number of patients opted to visit these centers instead of approaching SUMC directly. This likely reflects fears that the hospital, being the region’s only tertiary care center and a central institution in the conflict zone, would be overcrowded or potentially targeted by missile strikes. This behavioral shift underscores the importance of enhancing both the real and perceived safety of hospital environments and ensuring the resilience and accessibility of emergency medical services during wartime.

### Consistency and quality of hospital-based STEMI care

Despite these challenges, the study found that the quality of care provided at SUMC remained consistent. Clinical characteristics upon arrival and PCI outcomes did not differ significantly between the pre-war and war periods, indicating that healthcare providers at SUMC were able to maintain high standards of care even under extreme conditions. This resilience is commendable and suggests robust emergency preparedness and adaptability within the institution. This consistency in care could be attributed to the efficient triage strategy “Physician in triage”, ensuring that crucial time metrics such as D2B and P2B times were maintained effectively [13]. Well-coordinated triage and management strategies can significantly impact the timeliness and quality of acute cardiovascular care even in challenging environments [13].

### Equity in healthcare access

Additionally, the proportion of Bedouin patients presenting with STEMI remained stable across the study years, in contrast to other reports suggesting a decline in minority group hospital utilization during times of conflict [3]. This suggests that Bedouin Arabs felt secure and trusted the care provided at SUMC, despite the wartime conditions. Several factors may explain this phenomenon: SUMC has served for decades as the sole tertiary hospital in the Negev and is deeply embedded in the community it serves. Its staff is composed of both Jewish and Bedouin professionals, and the institution is known for providing culturally competent care. In addition, SUMC maintains longstanding collaborations with Bedouin community leaders, and its services are accessible in both Hebrew and Arabic. These factors likely contributed to patients’ confidence in seeking care without fear of discrimination, highlighting SUMC’s commitment to inclusive and equitable healthcare, even under conflict conditions.

### The role of public awareness and patient education

The study’s findings also emphasize the importance of educating patients about the necessity of seeking immediate medical attention for STEMI, regardless of ongoing conflicts. Public awareness campaigns and targeted education could help reduce the incidence of late arrivals and improve patient outcomes during wartime [14–16]. Examples of effective public awareness campaigns include utilizing social media platforms, local radio, and television broadcasts to disseminate information about the signs and symptoms of STEMI and the importance of seeking immediate medical care [17]. Community workshops and partnerships with local organizations could also enhance outreach efforts. Additionally, creating easily accessible online resources and hotlines can provide real-time support and information to individuals during conflicts [18].

### Stress, confounders, and the complexity of cardiovascular risk during conflict

Moreover, although the correlation analysis between the number of rockets launched and the number of STEMI cases and late arrivals was not statistically significant, this does not rule out a meaningful relationship. It is possible that chronic stress from prolonged exposure to a war environment—rather than acute rocket events—plays a more substantial role in elevating cardiovascular risk. Chronic psychological burden may lead to physiological dysregulation, including increased sympathetic activation, inflammation, and endothelial dysfunction, all of which are linked to myocardial infarction [19, 20]. Additionally, several unmeasured or uncontrolled confounding factors may have influenced the observed trends. These include reduced physical activity due to sheltering behaviors, changes in medication adherence, delays in prescription refills, limited access to primary care, or dietary changes during conflict [21, 22]. These complex and interacting factors underscore the need for further research, including studies designed to differentiate between the effects of acute versus chronic stressors. Prior research from other crisis settings, such as the September 11 attacks and the Bosnian War, demonstrated increased cardiovascular events associated with sustained psychological stress, even in the absence of direct physical exposure to violence [23, 24]. These findings support the hypothesis that the cumulative stress of living under threat may be more influential on cardiovascular outcomes than single events, and that future research should aim to disentangle these temporal and behavioral factors.

### Parallels with other crises: lessons from the COVID-19 pandemic

The challenges identified in this study resemble those seen during other large-scale crises, such as the COVID-19 pandemic, where delayed presentations of STEMI and reductions in ambulance use were also widely reported [25]. Both situations share common barriers—fear, uncertainty, and disrupted access to care—yet stem from different underlying causes: fear of viral exposure in one case, and fear of physical harm or perceived inaccessibility in the other. These similarities highlight the broader vulnerability of acute cardiovascular care during times of systemic disruption. Notably, crisis-response strategies successfully implemented during the pandemic—such as public campaigns emphasizing the urgency of symptoms [26], decentralized triage systems [27], and expanded use of telemedicine [28]—may offer valuable models for improving STEMI care during armed conflicts. Adapting such strategies to war zones, including reinforcing the message that hospitals remain open and safe, could help mitigate delays in care and reduce morbidity and mortality in similar future scenarios.

### Limitations

However, it is essential to acknowledge the limitations of this study. Firstly, the retrospective design may introduce selection bias and inaccuracies in medical record documentation. The study was conducted at a single center, which limits the generalizability of the findings to other settings. Additionally, the true reasons behind late arrivals remain unclear, necessitating further qualitative research to understand patient behavior and barriers to timely care during conflicts. Furthermore, the relatively short study period may not capture long-term outcomes and trends, which limits our understanding of the full impact of delayed treatment during conflicts.

## Conclusion

In conclusion, while hospital care remained robust, there was a marked increase in late arrivals and patients coming from emergency centers, indicating delays in seeking medical attention and fear of going directly to the hospital. These findings highlight the need for targeted patient education to ensure prompt care during conflicts and improve confidence in hospital safety and availability.

## Data Availability

According to the ethics committee, the data cannot be made available due to concerns regarding patient confidentiality. However, data may be made available upon reasonable request and subject to specific approval by the ethics committee. Requests for data access can be directed to Dr. Zeldetz, and will be reviewed in accordance with the ethical guidelines and institutional policies.

## Abbreviations

STEMI: ST-Elevation Myocardial Infarction
PCI: Percutaneous Coronary Intervention
SUMC: Soroka University Medical Center
LVF: Left Ventricular Function
VT/VF: Ventricular Tachycardia/Ventricular Fibrillation
CCI: Charlson Comorbidity Index
CIHD: Chronic Ischemic Heart Disease
DM: Diabetes Mellitus
HTN: Hypertension
ED: Emergency Department
ICCU: Intensive Cardiac Care Unit
FMC: First Medical Contact
P2D: Pain to Door
D2N: Door to Needle
D2B: Door to Balloon
P2B: Pain to Balloon
AdjOR: Adjusted Odds Ratio
SD: Standard Deviation
IQR: Interquartile Range
LOS: Length of Stay
